# Investigation of Micro-Bending of Sheet Metal Laminates by Laser-Driven Soft Punch in Warm Conditions

**DOI:** 10.3390/mi8070224

**Published:** 2017-07-18

**Authors:** Huixia Liu, Guoce Zhang, Zongbao Shen, Wenhao Zhang, Xiao Wang

**Affiliations:** School of Mechanical Engineering, Jiangsu University, Zhenjiang 212013, China; 2211503071@stmail.ujs.edu.cn (G.Z.); szb@ujs.edu.cn (Z.S.); Z10091122@163.com (W.Z.); wx@ujs.edu.cn (X.W.)

**Keywords:** micro-bending, sheet metal laminates, laser-driven soft punch, springback, element diffusion, warm conditions

## Abstract

Microscale laser dynamic flexible forming (µLDFF) is a novel ultrahigh strain rate manufacturing technology with high efficiency and low cost. However, the µLDFF is just confined to single-layer foil at present. In this work, sheet metal laminates (Cu/Ni) were selected as the experimental material for its excellent mechanical and functional properties, and a new micro-bending method of sheet metal laminates by laser-driven soft punch was proposed in warm conditions. The micro-mold and warm platform were designed to investigate the effects of temperature and energy on formability, which were characterized by forming accuracy, surface quality, element diffusion, and so on. The experimental results show that the forming accuracy and quality increased first and then decreased with laser energy, but the hardness increased consistently. In warm conditions, the fluidity of material was improved. The forming depth and accuracy increased for the relieved springback, and the surface quality increased first and then decreased. The tensile fracture disappeared with temperature for the decreased hardness and thinning ratio, and the element diffusion occurred. Overall, this study indicates that the formability can be improved in warm conditions and provides a basis for the investigation of micro-bending of sheet metal laminates by µLDFF in warm conditions.

## 1. Introduction

In recent years, the demand for micro-products has grown with the market of electronic products and micro-electro-mechanical systems, making precision and miniaturization an important direction of the manufacturing industry, and the research of micro-plastic forming technology become a hotspot. However, with the traditional micro-forming method it is difficult to meet industrial production demands, because of high cost, low processing efficiency and environmental pollution problems [1]. With the rapid development of laser technology, the microscale laser dynamic flexible forming (µLDFF) technique has gradually become an import dynamic high-pressure loading technology. The laser has many advantages, such as good repeatability, high accuracy and flexibility. Cheng et al. [2] had applied this technique to the micro-forming of mental foil. The results showed that high strain rate and formability was achieved by the laser-induced high-pressure shock wave forming process. Further research was conducted by Li et al. [3,4] to find the forming limits and fracture of the workpiece, which showed that formability increased compared with other forming processes of metal. Gao [5] studied the effect of laser shot peening on the fatigue properties of the workpiece. The results showed that laser shot peening causes a deeper compressive residual stress field, which can improve the fatigue strength of the material more effectively than the traditional surface strengthening method.

However, the direct µLDFF has a problem: the laser acts on the surface of the workpiece directly, which ablates the surface of the workpiece and destroys the surface quality [1]. In order to solve this problem and improve the formability of µLDFF, Wang et al. [1] had applied the laser-driven soft punch technique, a novel laser indirect shock forming method, to micro-forming of metal foil. In that process, low impedance medium flexible rubber was utilized as a soft punch, placed between the thin metal sheet and the black paint to protect the workpiece surface from being ablated. Liu et al. applied laser shock punch to fabricate micro-gears [6] and dish-shaped micro-parts [7], and found that soft punch with 200 µm in thickness is beneficial to homogenize energy and propagate the shock wave. It also has been found that the soft punch is favorable for increasing formability of the mental foils during the µLDFF, and the soft punch can easily separate from the workpiece after the forming process without excessive force [8].

It can be seen from above that the process for laser-driven soft punch forming mainly concentrates on a single-layer metal. However, with the development of industry, single-layer metals have been unable to meet the needs of industry. Therefore, many types of sheet metal laminates, composed of a plurality of layers of metal with dissimilar materials [9], have been produced and applied in micro-forming products, which need several different kinds of superior properties at the same time. The properties of laminates can be tailored by choosing proper layers and arrangements to improve its mechanical, thermal and electrical properties on demand [10]. Hence, the micro-forming of sheet metal laminates was studied. Hino et al. [11] investigated the springback of sheet metal laminates (Al/SS) in draw-bending with 1.5 mm thickness, and found that the springback was strongly affected by the strength difference between the component layers. Oya et al. [12] evaluated the formability of multilayer steel sheets with 1 mm thickness through tensile, V-bending and hemming tests. The laser bending of a SUS430/C11000/SUS430 laminated composite was investigated by Seyedkashi et al. [13] both experimentally and numerically. Recent studies have shown that many properties of material can be dramatically improved in terms of their laminated composites, including impact behavior, wear, corrosion, fracture toughness, fatigue behavior and damping capacity. In addition, the enhanced formability or ductility of brittle materials was also improved [14]. 

However, the forming behavior of sheet metal laminates is much more complicated than single-layer sheets because of dissimilar metal components. Therefore, it is important to find the technique of improving the formability to expand the application of sheet metal laminates. The laser-driven soft punch micro-bending method was applied in this paper for its advantages in improving the forming performance. In addition, a few recent studies have reported the improvement of forming performance at elevated temperature [15], which is called warm forming. However, the studies in warm conditions are just confined to the single layer at present. Ye, Cheng et al. [16] researched the formability of laser direct shock forming of the copper foil in warm conditions. The results showed that the forming depth can be increased by elevating forming temperature and laser energy. These effects on forming ability were also researched by Ma et al. [15] by charactering forming depth, forming accuracy and surface quality. It was found that the optimal forming temperature was appropriately 150 °C as the forming depth and forming accuracy was improved without deterioration of the surface integrity. Jiang et al. [17] investigated the influence of temperature on the deformability of pure copper, and found that forming temperature has important influence on the shape accuracy and metal flow behavior.

The main objective of this work is to investigate the forming performances of sheet metal laminates in warm conditions by laser-driven soft punch experiments, including the forming depth and accuracy, surface roughness and so on. Element diffusion was also involved. Influences included experimental temperature and laser energy. The micro-mold and warm platform were designed, and Cu/Ni laminated composite metal sheets were selected as the experimental material. The experiment found that formability was improved in warm conditions.

## 2. Experiment

### 2.1. Experimental Mechanism

**Figure 1:**
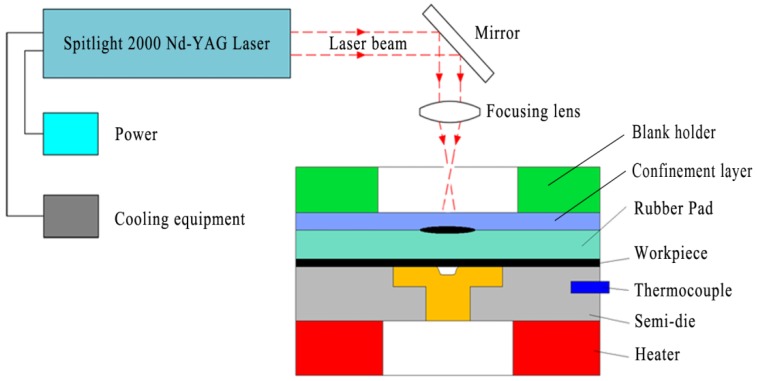
The schematic of μLDFF process.

Figure 1 illustrates the basic theory of μLDFF. The tooling set consists of confinement blank holder, confinement layer, black paint, soft punch, workpiece, rigid die, thermocouple and heater. The setup is workable for two conditions: (a) in cold conditions; (b) in warm conditions.

(a) In normal conditions: 

When the intense laser pulse penetrates the confinement layer and irradiates the black paint, the paint vaporizes into a high temperature and pressure plasma instantaneously. The plasma expands quickly and is limited by the confinement layer producing pressure within the soft punch. A strong elastic deformation of the soft punch occurred under the effect of the strong pressure. Due to the incompressible and hyperelastic characteristics of soft punch, the workpiece is deformed conformal to the underlying three-dimensional rigid die. 

(b) In warm conditions: 

The experimental theory of warm forming is similar to cold forming, but before the warm forming process, the workpiece and rigid die must be preheated to the set temperature by the warm system under the control of a PID controller, in which the thermocouple is used to monitor the real-time temperature of the experimental platform. The heater stops heat the platform while the temperature rises to the set temperature, and starts heat on the contrary. 

**Figure 2:**
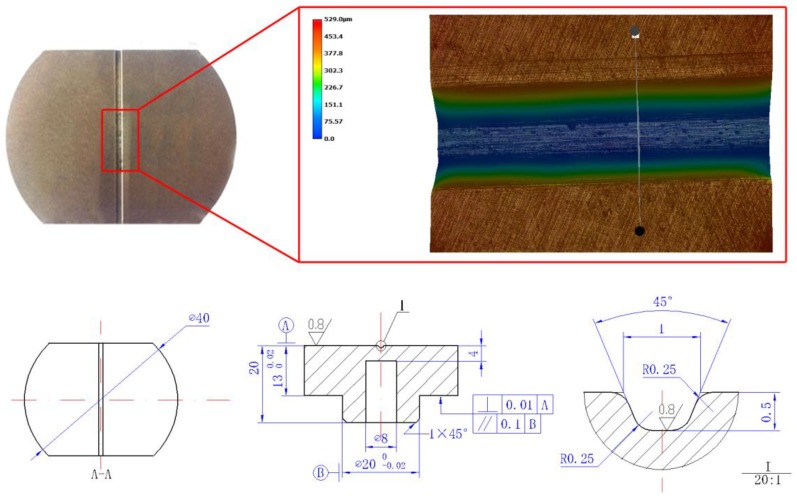
The graphic and engineering drawing of the rigid die.

The graphic and engineering drawing of the rigid die are shown in Figure 2. The graphic was observed using a KEYENCE VHX-1000C digital microscope. The stiffness plays an important role in maintaining the accuracy of the mold and workpiece during the forming process [18]. Therefore, the 1095 mold steel was chosen as the material to fabricate the micro-die for its great hardness, stiffness and good mechanical properties, to suffer the high shock pressure induced by the expansion of plasma, microgrooves of the die was fabricated by optic-curve grinding machine.

### 2.2. Materials Preparation

**Figure 3:**
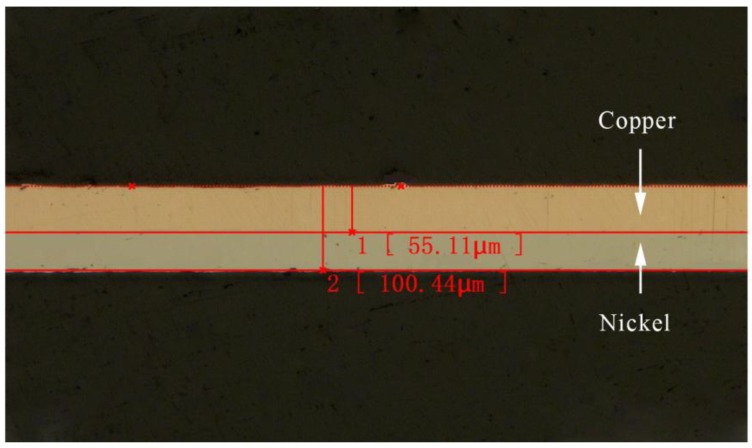
The structure of the Cu/Ni laminated composite metal sheets.

A laminated composite metal sheet, consisting of copper layer of 0.05 mm and pure nickel layer of 0.05 mm in thickness, was used in the experiment. The material was fabricated by press and cold roll bonding process. The structure of the laminated composite metal is shown in Figure 3, and the mechanical properties of each layer are listed in Table 1 [19].

According to the research of Yilamu K et al. [20], the relative position of the weak and the strong layers of the sheet metal laminates has a great influence on formability. Therefore, in the experiment, the composite material was placed under the way that the copper layer (weak layer) was the impacted layer and the nickel layer (strong layer) was the bottom layer.

### 2.3. Parameter Design

The laser employed in the experiment was Spitlight 2000 Nd:YAG (InnoLas Corporation, Krailling, Germany) with Gaussian distribution beam, and its main parameters is listed in Table 2. In order to get the desired spot diameter (about 2 mm), the defocusing was selected as −10 mm [21]. Because of the high temperature and shock impedance, K9 glass was selected as the transparent confining layer, with the diameter of 50 mm and the thickness of 4 mm, to constrain the expansion of plasma. Four experimental temperatures and five laser energies were selected, and the details of experimental parameters are shown in Table 3.

## 3. Results and Discussion

### 3.1. Forming Depth

**Figure 4:**
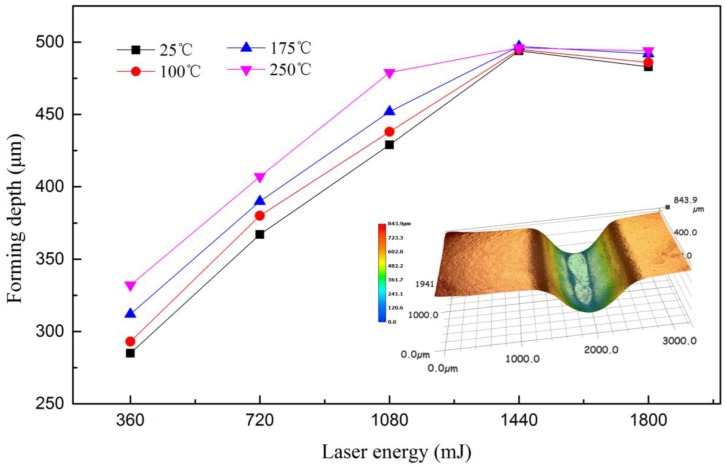
Influence of laser energy and temperature on forming depth.

**Figure 5:**
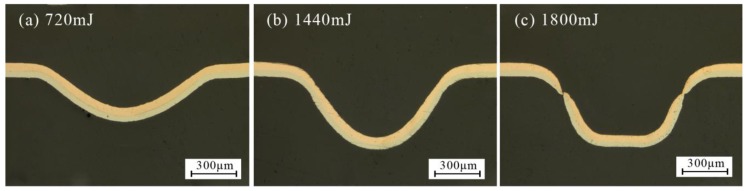
Three profiles of different forming depths at 25 °C and different laser energies: (**a**) 720 mJ, (**b**) 1440 mJ and (**c**) 1800 mJ.

A series of experiments were conducted in warm conditions and the maximum value was defined as the forming depth at the bottom of the valley of the formed workpiece. The results are shown in Figure 4. It was found that at room temperature (25 °C), the forming depth increased first and then decreased with laser energy, and the largest forming depth was achieved at 1440 mJ. When the laser energy reached 1440 mJ, the workpiece contacted with the bottom of the rigid die, and no longer increased, which is related to the research of Zhang et al. [22]; when the energy was 1800 mJ, the springback of workpiece occurred because of the intense collision between the workpiece and rigid die, so the forming depth decreased, as shown in Figure 5.

The springback can be explained as follows: First, the silicone rubber would return to the initial shape quickly after the maximum elastic deformation. However, at some parts, the plastic deformation was not complete, within the elastic deformation or between the elastic and plastic deformation; second, as the laser impact micro-forming is a rapid prototyping process, the forming depth increased with the energy and sufficient impact pressure. While the workpiece contacted with the mold, severe plastic deformation occurred with different speeds. When the surrounding has pasted the mold completely, the workpiece collided with the rigid die, resulting in springback.

At warm conditions, the forming depth increased with temperature. By elevating the temperature from 25 °C to 250 °C, an increase of over 47 μm in forming depth was achieved, approximately 16.5%. The improved forming depth can be explained by the increased ductility and reduced flow stress. However, when the laser energy reached 1440 mJ, the temperature has little effect on the forming depth, because the workpiece contacted with the rigid die. When the laser energy changed from 1440 mJ to 1800 mJ, the depth at warm conditions was higher than that at room temperature, and the collision between the workpiece and rigid die became more moderate because the increase of temperature made the fluidity of the workpiece increased, so the springback increased too. 

### 3.2. Surface Quality

It is well known that the deterioration of surface integrity can possibly lead to the failure of material. Therefore, it is necessary to research the effects on surface quality of a workpiece in warm conditions. Axio CSM 700 was employed to obtain the surface morphology and roughness of the rigid die and formed parts. 

**Figure 6:**
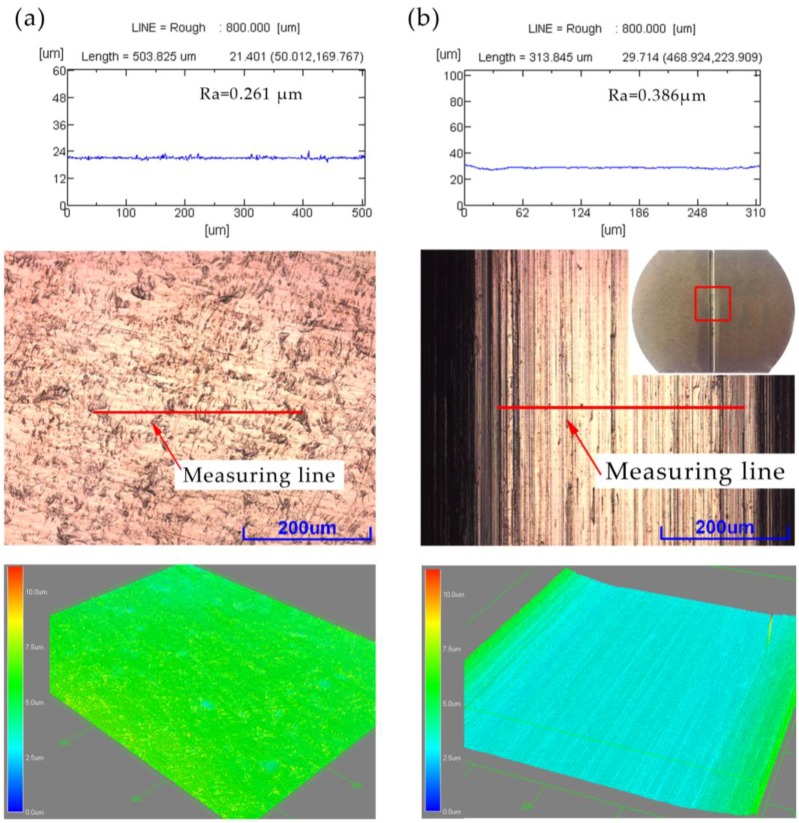
The surface roughness: of (**a**) raw material nickel and (**b**) rigid die.

The value Ra of the formed region was measured at the bottom of the valley of the formed part to evaluate the surface roughness. The value of Ra along the length or width of the groove is different. The mold was machined along the length of the groove, a small protrusion along this direction of mold, so that the roughness variation measured along the groove is small, and the roughness level of the entire bottom of the workpiece cannot be well reflected. Therefore, the direction perpendicular to the groove was adopted in this paper, and the measurement results are accurate. Figure 6 shows the surface roughness of raw material nickel and the rigid die. The results showed the roughness of the rigid die and nickel was 0.386 μm and 0.261 μm, respectively.

**Figure 7:**
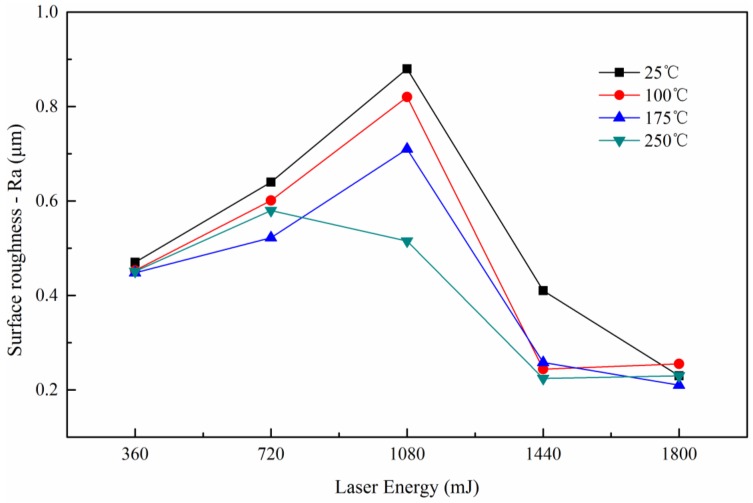
Influences of forming temperature and laser energy to surface roughness.

**Figure 8:**
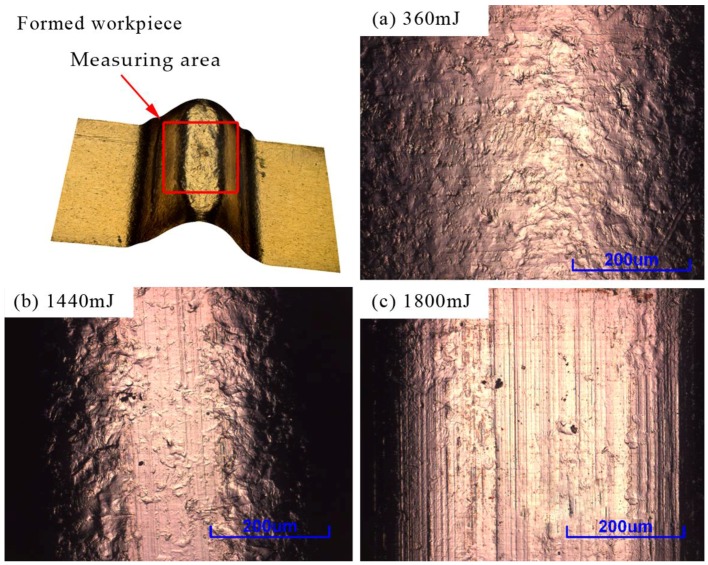
Three different forms of forming process at different laser energy: (**a**) 360 mJ, (**b**) 1440 mJ and (**c**) 1800 mJ.

Figure 7 shows the roughness of the formed parts at four temperatures. It was observed that the surface roughness showed a trend that increased first and then decreased with the laser energy. Because there were lines on the surface during the rolling process, and in laser shock process, the workpiece was deformed in all directions, making the lines and roughness become larger and larger. With the increase of laser energy, the plastic deformation of the specimen was strengthened, making the grain size of the workpiece surface roughed and leading to the increase of the surface roughness of the formed parts [23], as shown in Figure 8a. With further increase of energy, the workpiece copied the die lines after contacted with the rigid die, so the roughness was reduced, as shown in Figure 8b. When the energy reached 1800 mJ, the workpiece completely pasted the rigid die, and copied the lines, making the roughness of workpiece similar with the die, about 0.386 μm, as shown in Figure 8c. 

It can also be found from Figure 7 that the surface roughness decreased with the forming temperature. This is probably because, the fluidity and plasticity of the material was improved and the impact of line on the forming parts was alleviated, so the friction force of the workpiece between the soft punch and rigid die was reduced. Overall, the surface roughness was reduced after the forming process in warm conditions.

When the laser energy was 1080 mJ, the surface roughness increased first and then decreased. This is because when the temperature was 250 °C, the workpiece contacted with the rigid die. Finally, the surface roughness showed no obvious difference for the specimens with different temperature when the laser energy was 1800 mJ, the final roughness of the bottom mainly depended on the surface roughness of the die.

### 3.3. Thickness Distribution

**Figure 9:**
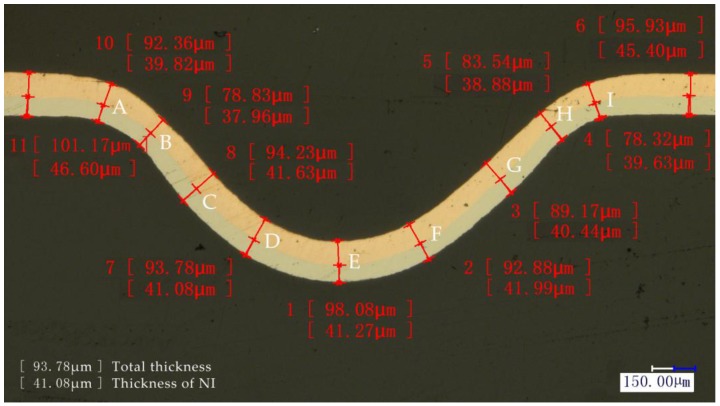
Measurement locations of thickness distribution.

The thickness thinning ratio of the formed parts is a significant forming property in the micro-forming process. Severe thinning of the formed parts led to compressive stress, localized necking, and even failure of the component. Therefore, the thinning ratio was characterized by using the cold-mounted process in this paper, and the mounted workpiece was grinded with 80# to 3000# sand paper and then polished. Finally, the thickness was measured as shown in Figure 9.

**Figure 10:**
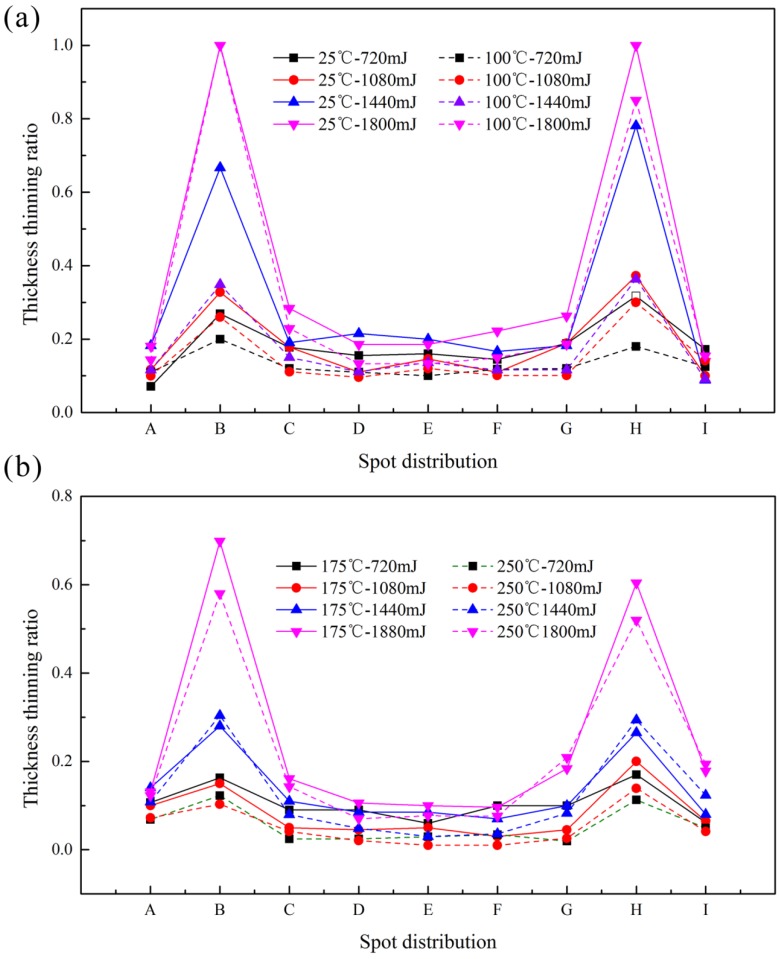
The distribution of thickness thinning ratio with different laser energy and temperature: (**a**) at 25 °C and 100 °C, (**b**) at 175 °C and 250 °C.

The thinning ratio of the sheet and each layer was calculated respectively by Equation (1). Where $t_{0}$ and $t_{i}$ are the thickness before and after deformation respectively. The distribution of thickness thinning ratio with different laser energies is shown in Figure 10.

(1) $$T=\frac{t_{0}-t_{i}}{t_{0}}\times 100\%\;,\;i=1,2,3\ldots 9$$

It can be seen from Figure 10 that the thinning ratio and magnitude fluctuation were small with low laser energy, and it increased with the laser energy. The maximum value was achieved at the points B and H, and even reached 1, which meant the fracture occurred. The appearance of this phenomenon is because of the short time of laser action, and the material at the chamfer did not have enough time to flow into the mold cavity. In the mold cavity, the workpiece was bent depending on the initial elastic deformation of silicone rubber. Therefore, the deformation is small.

**Figure 11:**
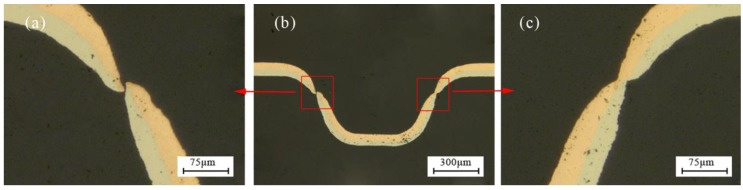
The necking and fracture of the workpiece: (**a**) fracture, (**b**) the overall, and (**c**) necking.

With the increase of laser energy, necking occurred in the chamfer area. Then the workpiece was squeezed into the mold cavity by the silicone rubber, making the thickness further reduced to complete the plastic deformation, the distribution was no longer uniform. When the energy reached 1800 mJ, due to the smaller thickness, the smaller number of grains was involved in deformation, and the workpiece has friction with the soft punch and rigid die, so poor mobility caused the uneven deformation, even fracture, which was the critical load point, as shown in Figure 11.

**Figure 12:**
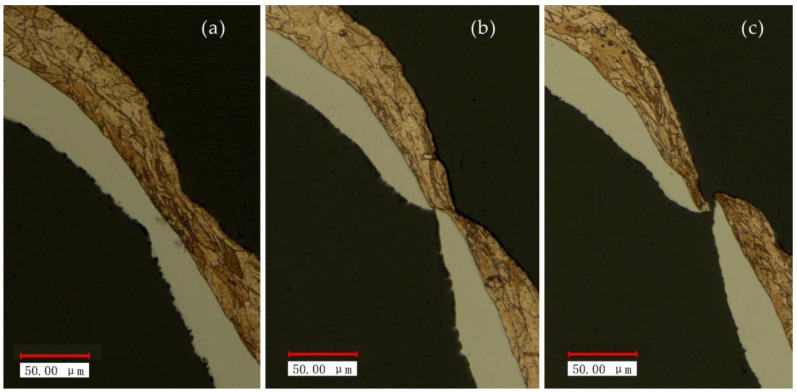
The microstructure of the fracture process at 25 °C and different energy: (**a**) 720 mJ, (**b**) 1080 mJ and (**c**) 1800 mJ.

In order to verify the above reasons, the microstructure of the fracture process was observed. The metallographic etchant was prepared by the mass of 5 g FeCl_3_, 15 mL HCl and 85 mL H_2_O. The polished samples were immersed in the etching solution for 2 to 3 s. The microstructure achieved after etching was shown in Figure 12. It was can be found that with the increase in energy, due to the friction and poor mobility, the grains were elongated. When the tension is greater than the binding force between the grains, fracture occurred.

**Figure 13:**
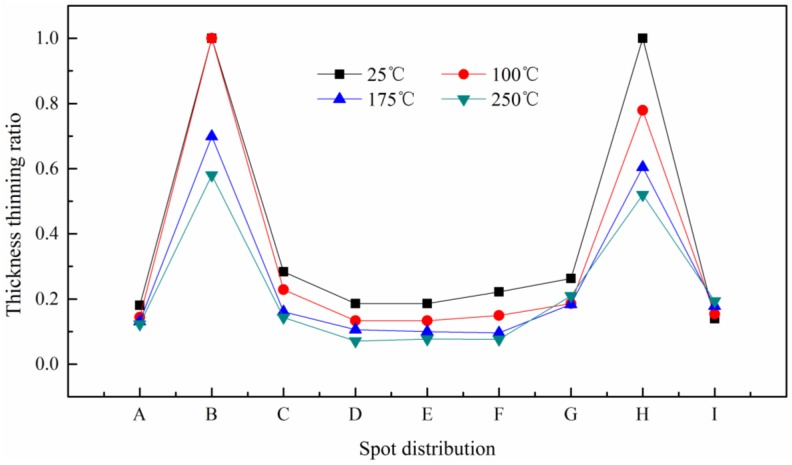
The distribution of thickness thinning ratio with different temperature at 1800 mJ.

**Figure 14:**
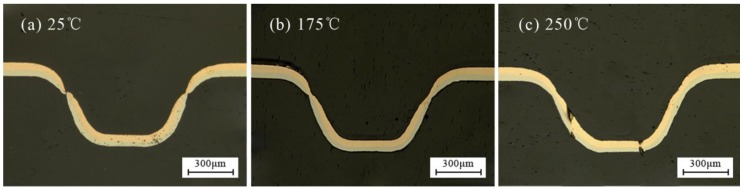
The change process of thickness thinning ratio with temperature at 1800 mJ: (**a**) 25 °C, (**b**) 175 °C and (**c**) 250 °C.

Figure 13 shows the distribution of thinning ratio with temperature. The maximum value was found at the critical load point, where the fracture occurred, as shown in Figure 14a. The fracture disappeared with the increase of temperature to 175 °C, as shown in Figure 14b. The necking was also alleviated by further reduced of thinning ratio with the increase of temperature to 250 °C. This is because of higher fracture strain, better ductility and improved plasticity of material compared with room temperature (25 °C), as shown in Figure 14c. 

**Figure 15:**
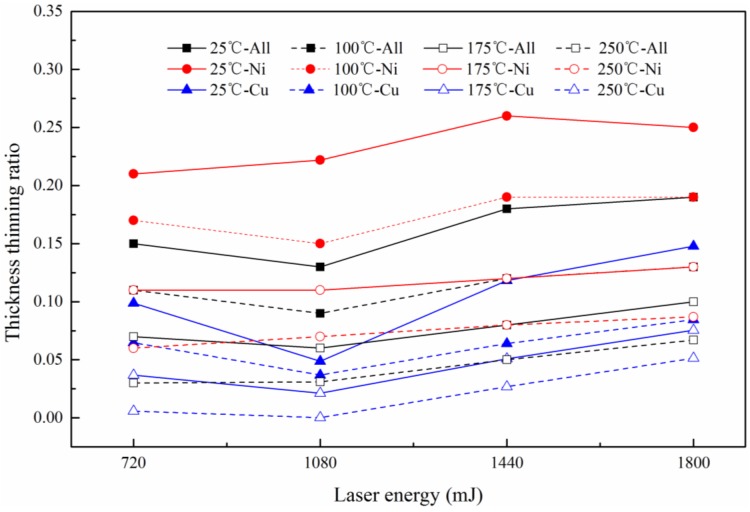
The thinning ratio distribution of copper, nickel and composite layer.

In order to further understand the effects on thinning ratio of sheet metal laminates, the thinning ratio of each layer was also investigated. Figure 15 shows the thinning ratio distribution of copper, nickel and composite layer, respectively. It can be seen that the thinning ratio of copper was generally smaller than nickel under the same condition, but this trend gradually became less apparent with the temperature.

**Figure 16:**
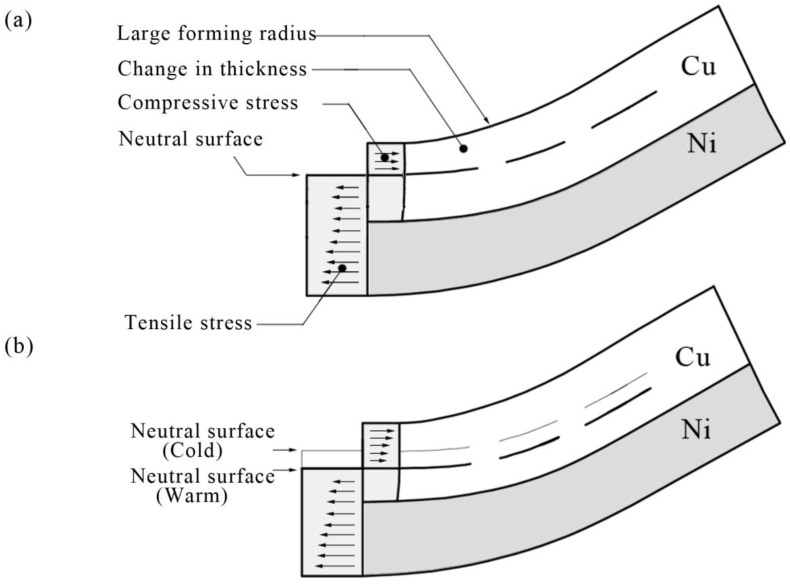
Schematic illustration of mechanism of changes in thickness: (**a**) at 25 °C, (**b**) at 250 °C.

This phenomenon can be related to the performance of the materials: the hardness of copper is less than nickel. In this experiment, when the workpiece was bent, the compressive stress was generated on the copper surface and the interface of copper and nickel, the tensile stress was generated on the nickel surface, as shown in Figure 16a. This was similar with the research of Yilamu et al. [20]. However, in this paper, the neutral lay in the weak layer, which was different from the research of Yilamu. This was because unlike the traditional pressure bending process, during the µLDFF process, all the forming areas of the workpiece were subjected to the impact pressure that produced by the plasma explosion after the laser shock, resulting in the thickness of all forming areas were reduced. With the increase of temperature, the two layers’ deformation became more uniform. This was because, the neutral layer moved toward the side of nickel layer with the temperature, as shown in Figure 16b. Which resulting in the tensile stress of the two materials decreased, and the compressive stress increased. Therefore, the gap of thinning ratio between copper and nickel gradually became less apparent with the temperature.

### 3.4. Forming Accuracy

**Figure 17:**
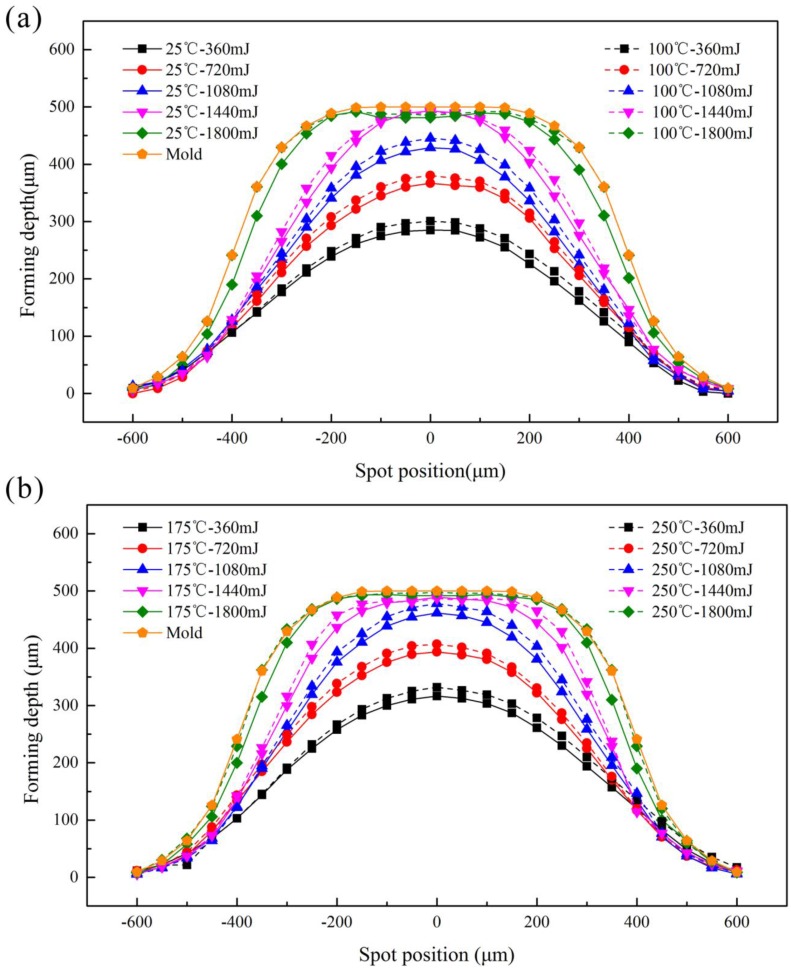
Effects of laser energy on the forming symmetry at different temperature: (**a**) at 25 °C and 100 °C, (**b**) at 175 °C and 250 °C.

Fitability and symmetry are the aspects to characterize the formability of accuracy, which is the ability of workpiece to obtain the shape of the rigid die during the forming process. In this paper, the 2D curves in different conditions were investigated to research the forming accuracy in Figure 17. The dotted and solid lines in this figure represent the experimental data at different temperature, and the outside solid line is the contour curve of the rigid die. The contours of the formed parts should be symmetrical along the X axis zero because the rigid die is symmetrical and the laser employed in the experiment with Gaussian distribution beam. From Section 3.3, it was known that with the increase of temperature, the material has higher fracture strain, better ductility and plasticity, the deformation were more uniform and forming ability was improved. Therefore, the contour aligned along the X axis zero better after the forming process with higher temperature. 

**Figure 18:**
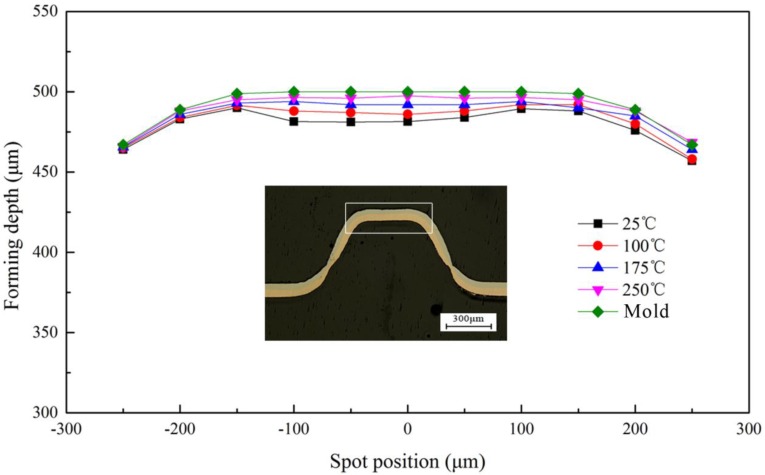
The effects of temperature on the fitability at 1800 mJ.

When the laser energy was 1800 mJ, the regular pattern was still valid. This was because the workpiece had reached the maximum forming depth. However, the workpiece and the rigid die were not completely conformed at 25 °C for the springback; when the temperature is 250 °C, the fluidity increased and the springback decreased, resulting in the better fit between the workpiece and rigid die. In order to research the effect of springback on the fitability at different temperatures, the next experiment about fitability was conducted at 1800 mJ, as shown in Figure 18.

According to Section 3.1, when the energy was 1800 mJ, the springback of workpiece occurred after contacted with the rigid die, but the increase of temperature making the fluidity of the material increased, the forming depth also increased for the reduce of springback. The fitability of the workpiece was improved. In summary, with the increase of temperature, the fitability and the symmetry improved, the forming accuracy became better and better.

### 3.5. Hardness Distribution

**Figure 19:**
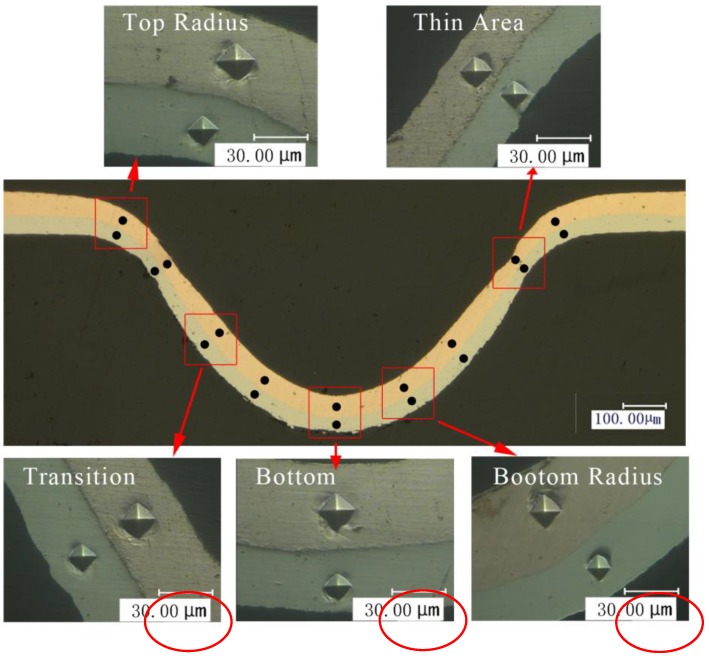
The distribution of micro-hardness test points.

It is well known that hardness is directly related to the strength of metals [24]. In order to explore the mechanical property of Cu/Ni laminated metal sheet before and after micro-forming, micro-hardness was measured. In this paper, the microscopic indentation experiment mainly revealed the distribution of micro-hardness of the cross section of the forming parts, and discussed the influences of the laser energy and temperature on it. The maximum load used in the experiment was 245 mN and was maintained for 10 s when the loading force was reached and then unloaded. Nine positions of the cross section were selected as the micro-indentation test points, as shown in Figure 19.

**Figure 20:**
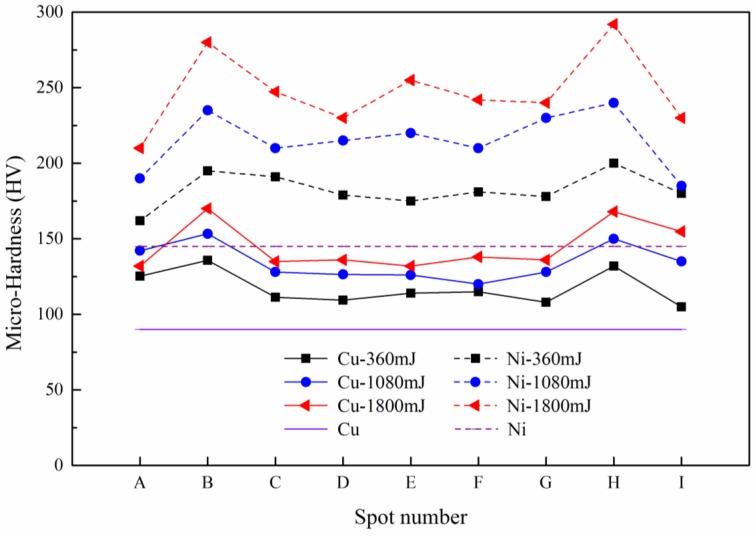
The micro-hardness distribution at room temperature.

**Figure 21:**
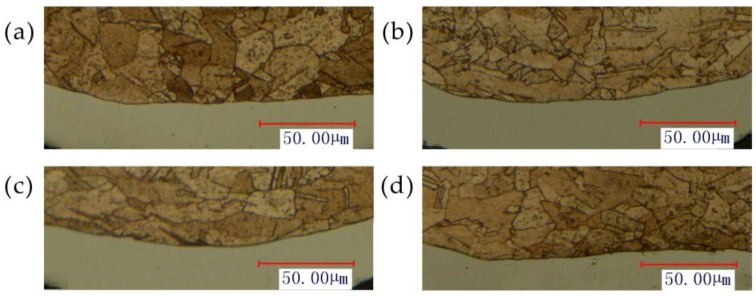
The microstructure at 25 °C and different energy: (**a**) 720 mJ, (**b**) 1080 mJ, (**c**) 1440 mJ and (**d**) 1800 mJ.

The hardness results of Cu/Ni at room temperature are shown in Figure 20. It can be seen that the hardness of Cu and Ni are both improved: compared with unformed parts, the hardness of Cu and Ni improved about 20% under the laser shock at 360 mJ. The tendency of experimental results in agreement with Cheng et al. [2]: the hardness of the workpiece increased by 6 to 8 times after the dynamic impact of the laser. Further increased the laser energy, the hardness of Cu and Ni increased, and the hardness value of point B and H were the largest, which was similar with the distribution of thinning ratio in Figure 10. This was because in cold process, the greater plastic deformation, the higher local hardness [25]: It can be found in Figure 11 and Figure 20 that, with the increase of energy, plastic deformation increased. The workpiece were subjected to tensile or compressive stress, causing strain hardening in these areas. In addition, the µLDFF was adiabatic high strain rate plastic deformation, which making the microstructure of the workpiece was refined, mechanical properties was also been improved. Contrast Figure 12 and Figure 21, it can be found that the filaments at the fillet were subjected to more tensile stress, and local plastic strain occurred. So the hardness of the workpiece at the fillet was greater than at the bottom of the workpiece.

**Figure 22:**
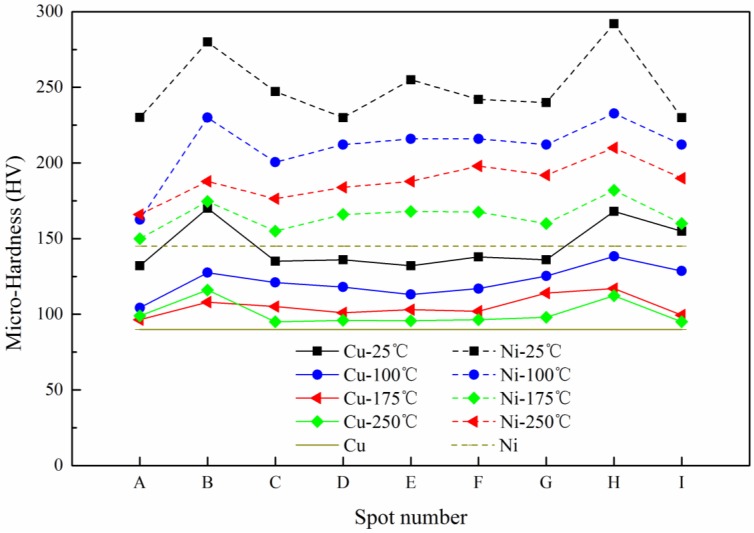
The micro-hardness distribution of Cu and Ni at 1800 mJ.

**Figure 23:**
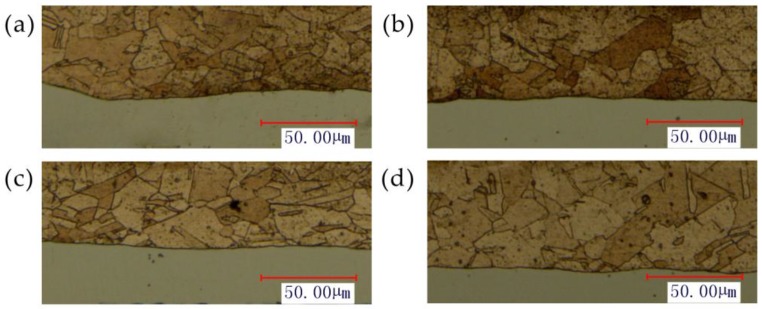
The microstructure at 1800 mJ and different temperature: (**a**) 25 °C, (**b**) 100 °C, (**c**) 175 °C and (**d**) 250 °C.

In order to understand the effect of temperature on the hardness, the variation of Cu and Ni hardness at different temperatures were investigated at 1800 mJ. The experimental results are shown in Figure 22. It can be seen that after adding the temperature, the hardness of Cu and Ni showed a downward trend. The reason can be surmised that with the increases of temperature, the fluidity of material was improved, and the material gradually softened. It can be seen from Figure 23 that the grain size increases slightly with temperature, the number of grain boundaries decreased, grain boundary strengthening effect is weakened. Therefore, the surface hardness of workpiece was decreased. As the temperature continued to increase, the ratio of decrease of hardness slowed down, and the hardness of points B and H were consistent with other points. This was similar to Figure 13, where the thinning ratio varied with temperature. This was because with the increase of temperature, the material became soft, and the plastic deformation decreased. Overall the forming became more uniform, and the hardness of the material decreased in warm conditions.

### 3.6. Element Diffusion

Interface bonding strength is an important indicator to evaluate the performance of metal. In this investigation, two layers of the workpiece, fabricated by press and cold roll bonding, bite each other to form a mechanical combination because of the friction between two layers, but this combination of strength is relatively low. According to the research of Jain et al. [26], after annealed, the metal compound produced by the diffusion of elements will be formed on the interface of the sheet metal laminates, which realized the excess of metal from mechanical bonding to metallurgy bonding strength of the bonding surface. 

**Figure 24:**
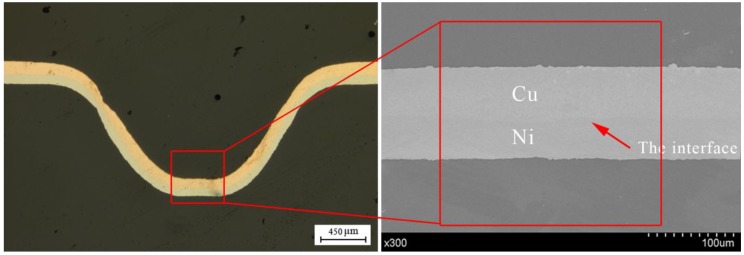
The position of the interface measured by SEM.

**Figure 25:**
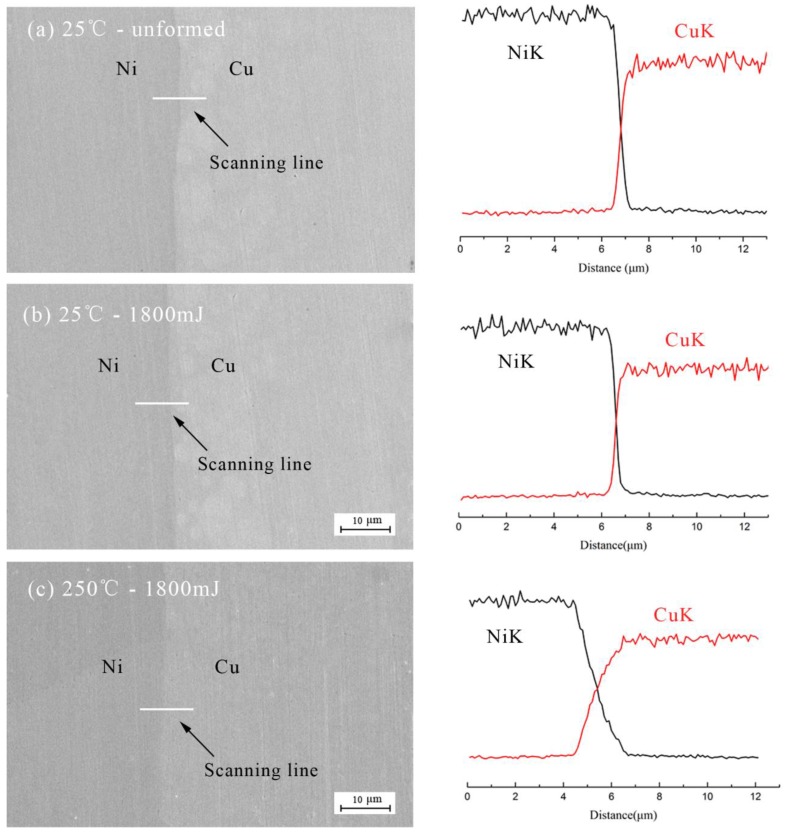
The results of EDX line scan: (**a**) unformed, (**b**) at 25 °C and (**c**) at 250 °C.

In this paper, different energy and temperature were used as factors to study the effects on the diffusion of Cu/Ni, the analysis of diffusion in Figure 24 was carried. The results were obtained by EDX line scan analysis of a 12 μm line perpendicular to the Cu/Ni interface and the results are shown in Figure 25. The “NiK” and “CuK” mean the K line of nickel and copper, respectively. The black and red curves indicate the changes of element content of Ni and Cu, respectively. 

Figure 25a,b show the composition profile across the Cu/Ni interface before and after forming process at 25 °C, respectively. It can be seen that element content (Cu and Ni) varied sharply in the weld interface. The results showed that apparent element diffusion did not occurr across the weld interface with increased energy [27]. This was because no severe plastic deformation was imposed during the forming process. According to the deformation induced inter-diffusion process [28,29], no intermetallic phase was formed in the Cu/Ni interface with no diffusion of the two elements.

Figure 25b,c show the composition profile before and after warm forming process at 1800 mJ, respectively. The diffusion slope in Figure 25c was smaller than that in Figure 25b, which indicated that intermixing of the two elements at the interface has already occurred, and intermetallic phase islands grow along interface by the diffusion of the two elements with temperature [26]. This may be because with the increase of the forming temperature, the energy of the atoms in the copper and nickel element increase and the motion is more active, so that the stability of the element decreases with the increase of the temperature, and the diffusion between the elements becomes easier. In addition, as the temperature increases further, this trend will increase, that is, the diffusion will be strengthened and the thickness of the intermediate layer will increase [30]. However, due to differences in the energy conditions of the atoms on the surface, no significant compound layer was formed, that is, the interface between the two elements did not changed significantly of the interface, the affected area of the bonding zone only about 2 μm. Which can be found by compared the graphics in Figure 25b,c.

## 4. Conclusions

For the laser-driven soft punch micro-forming technology, this paper proposes sheet metal laminates at different laser energies and temperatures to investigate the formability. The conclusions are summarized as follows:

1. At room temperature (25 °C), a springback appeared due to the large energy, and the forming depth increased first and then decreased with laser energy; the fluidity of the material was improved and the forming depth increased with temperature, but the ratio of the increase slowed down.
2. With the increase of laser energy, the roughness of the workpiece increased first and then decreased for the springback. In addition, the roughness reduced with the increase of temperature. Therefore, 250 °C is the optimal forming temperature.
3. The thinning ratio increased with the laser energy, and the maximum value was achieved under the rounded corners, where tensile fracture occurred. With the increase of temperature, the ratio decreased, and the fracture disappeared. The neutral layer moved to near nickel side.
4. The forming accuracy was determined by the forming depth, so the trend of the change of forming accuracy was similar to the forming depth. At room temperature, the fitability was improved by laser energy, but with the existence of springback, the further increase of fitability declined; with the increase of temperature, the springback was relieved, the fitability and symmetry were improved, so the forming accuracy was improved.
5. The hardness of nickel and copper were measured at the same position with the thinning ratio. It was found that the distribution of hardness was similar to the distribution of thickness with laser energy and temperature, which reflected the effect of plastic deformation on hardness.
6. With the increase of laser energy, almost no element diffusion occurred between the copper and nickel. However, the diffusion of elements occurred with temperature, and the interfacial bonding strength of the sheet metal laminates increased, even though the diffusion was small, and only a few intermetallic compounds were produced.

The forming results indicated that heating-assisted laser-driven soft punch micro-forming would be a good choice to improve the micro-forming abilities of sheet metal laminates. In addition, further intensive work needs to be done in future, which investigates the microstructure and size effect of the sample. In addition, simulation will also be involved to verify the experimental conclusions.

## Figures and Tables

**Table 1:** The mechanical properties of copper and pure nickel.

Material	A (MPa)	B (MPa)	Young’s Modulus (GPa)	Poison’s Ratio	n	C
Copper	90.0	292.0	124	0.34	0.310	0.025
Pure nickel	163.0	648.0	207	0.31	0.330	0.006

**Table 2:** Main parameters of Spitlight 2000 Nd:YAG Laser.

Parameters	Values
Pulse energy	80–1800 mJ
Wave length	1064 nm
Pulse width	8 ns
Energy stability	<±1%
Exit spot diameter	9 mm

**Table 3:** The experimental parameters.

Parameters	Values				
Temperature	25 °C	100 °C	175 °C	250 °C	--
Laser energy	20%	40%	60%	80%	100%

## References

[1] Wang X., Du D.Z. (2013). Investigation of microscale laser dynamic flexible forming process—Simulation and experiments. Int. J. Mach. Tools Manuf..

[2] Cheng G.J., Pirzada D. (2007). Microstructure and mechanical property characterizations of metal foil after microscale laser dynamic forming. J. Appl. Phys..

[3] Li J., Gao H. (2010). Forming limit and fracture mode of microscale laser dynamic forming. J. Manuf. Sci. Eng..

[4] Li J., Cheng G.J. (2010). Multiple-pulse laser dynamic forming of metallic thin films for microscale three dimensional shapes. J. Appl. Phys..

[5] Gao Y.K. (2011). Improvement of fatigue property in 7050–T7451 aluminum alloy by laser peening and shot peening. Mater. Sci. Eng. A.

[6] Liu H.X., Li J.W. (2015). Experimental and numerical simulation research on micro-gears fabrication by laser shock punching process. Micromachines.

[7] Liu H.X., Sha C.F. (2016). Fabrication of Dish-Shaped Micro Parts by Laser Indirect Shocking Compound Process. Micromachines.

[8] Rhim S.H., Son Y.K. (2005). Punching of ultra small size hole array. CIRP Ann. Manuf. Technol..

[9] Kim J.K., Yu T.X. (1997). Forming and failure behaviour of coated, laminated and sandwiched sheet metals: A review. J. Mater. Process. Technol..

[10] Lee T.H., Lee Y.J. (2015). Mechanical and asymmetrical thermal properties of Al/Cu composite fabricated by repeated hydrostatic extrusion process. Met. Mater. Int..

[11] Hino R., Goto Y. (2003). Springback of sheet metal laminates in draw-bending. J. Mater. Process. Technol..

[12] Oya T., Tiesler N. (2010). Experimental and numerical analysis of multilayered steel sheets upon bending. J. Mater. Process. Technol..

[13] Seyedkashi S.H., Gollo M.H. (2016). Laser bendability of SUS430/C11000/SUS430 laminated composite and its constituent layers. Met. Mater. Int..

[14] Tayyebi M., Eghbali B. (2013). Study on the microstructure and mechanical properties of multilayer Cu/Ni composite processed by accumulative roll bonding. Mater. Sci. Eng. A.

[15] Ma Y.J., Wang X. (2014). Research on Laser-driven Flyer Microforming in Warm Condition. Key Eng. Mater..

[16] Ye C., Cheng G.J. (2010). Effects of temperature on laser shock induced plastic deformation: The case of copper. J. Manuf. Sci. Eng..

[17] Jiang Z.Y., Zhao J.W. (2016). Influences of temperature and grain size on the material deformability in microforming process. Int. J. Mater. Form..

[18] Del Pozo D., López de Lacalle L.N. (2008). Prediction of press/die deformation for an accurate manufacturing of drawing dies. Int. J. Adv. Manuf. Technol..

[19] Bae G., Xiong Y. (2008). General aspects of interface bonding in kinetic sprayed coatings. Acta Mater..

[20] Yilamu K., Hino R. (2010). Air bending and springback of stainless steel clad aluminum sheet. J. Mater. Process. Technol..

[21] Wang X., Yuan Y.Q. (2014). Investigation of the forming pressure and formability of metal foil by laser-driven multi-layered flyer. Opt. Laser Technol..

[22] Zhang X.Q., Zhang Y. (2016). Numerical and experimental investigations of laser shock forming aluminum alloy sheet with mold. Int. J. Mater. Form..

[23] Choi Y.S., Piehler H.R. (2004). Formation of mesoscale roughening in 6022-T4 Al sheets deformed in plane-strain tension. Metall. Mater. Trans. A.

[24] Wang X., Ma Y.J. (2015). Size effects on formability in microscale laser dynamic forming of copper foil. J. Mater. Process. Technol..

[25] Sonmez F.O., Demir A. (2007). Analytical relations between hardness and strain for cold formed parts. J. Mater. Process. Technol..

[26] Jain M., Gupta S.P. (2003). Formation of intermetallic compounds in the Ni–Al–Si ternary system. Mater. Charact..

[27] Mozaffari A., Manesh H.D. (2010). Evaluation of mechanical properties and structure of multilayered Al/Ni composites produced by accumulative roll bonding (ARB) process. J. Alloys Compd..

[28] Sauvage X., Dinda G.P. (2007). Non-equilibrium intermixing and phase transformation in severely deformed Al/Ni multilayers. Scr. Mater..

[29] Hebert R.J., Perepezko J.H. (2004). Deformation-induced synthesis and structural transformations of metallic multilayers. Scr. Mater..

[30] Mozaffari A., Hosseini M. (2011). Al/Ni metal intermetallic composite produced by accumulative roll bonding and reaction annealing. J. Alloys Compd..

